# Re-expansion pulmonary edema after routine use of cardiopulmonary bypass in cardiac surgery: Case report

**DOI:** 10.5339/qmj.2025.61

**Published:** 2025-06-09

**Authors:** Abdulaziz Alkhulaifi, Bassam Shoman, Adnan Saadeddin, Shady Ashraf Mohammed, Hafeez Lone, Maurice Maksood

**Affiliations:** 1 Department of Cardiothoracic Surgery, Hamad Medical Corporation, Doha, Qatar; 2 Department of Cardiothoracic Anesthesia, Hamad Medical Corporation, Doha, Qatar *Email: saadeddinmd@gmail.com

**Keywords:** Re-expansion pulmonary edema, pulmonary edema, cardiopulmonary bypass, coronary artery bypass grafting, cardiac surgery, postoperative pulmonary complications

## Abstract

**Background:**

Re-expansion pulmonary edema (REPE) is traditionally associated with the resolution of pneumothorax or pleural effusion. Its occurrence after routine cardiopulmonary bypass (CPB) in cardiac surgery is rare. The incidence of REPE after treatment of pneumothorax or pleural effusion is less than 1%, but it carries a mortality rate of up to 20%.

**Case Presentation:**

We present a case of REPE in a 64-year-old male undergoing elective coronary artery bypass grafting. Despite an uneventful surgery and standard perioperative management, the patient developed REPE, manifested with increased airway pressures, blood-tinged secretions, and compromised oxygenation post-CPB. Immediate intervention comprising mechanical ventilation adjustments, diuretics, and vasopressor support was initiated to facilitate recovery. The pulmonary edema resolved within 24 hours after the surgery, and the patient was transferred to the surgical high-dependency unit (HDU) on the third postoperative day.

**Discussion:**

This case reports a rare occurrence of REPE following routine CPB and highlights the multifactorial pathogenesis involving reperfusion injury and altered pulmonary physiology. Possible mechanisms include reperfusion injury from free radicals, cytokine release, and increased vascular permeability. The management of REPE requires prompt recognition and treatment and involves diuretics, ventilatory adjustments, and hemodynamic monitoring.

**Conclusion:**

REPE, though rare post-CPB, requires a high index of suspicion and prompt management to prevent adverse outcomes.

## Introduction

Re-expansion pulmonary edema (REPE) following cardiac surgery is a clinically significant complication. It usually occurs after minimally invasive cardiac surgery, as well as following lung expansion after the resolution of pneumothorax or drainage of pleural effusion.^1^

The etiology is multifactorial and may include mechanical, hemodynamic, and biochemical factors.^2,3^ However, its occurrence during routine cardiac surgery with cardiopulmonary bypass (CPB) is exceedingly rare and not well-documented.^1^ We describe a case of REPE in the setting of open-heart surgery. This case was managed in the heart hospital of Hamad Medical Corporation in Qatar, a tertiary governmental hospital specializing in adult cardiology and cardiac surgery. Written consent was obtained from the patient, and the Medical Research Center (MRC) granted approval for the case report to be published (MRC no. 0424298).

## Case Presentation

A 64-year-old male patient, non-smoker, diabetic (type 2), hypertensive, with mild chronic kidney impairment, was diagnosed with coronary artery disease (CAD). He underwent percutaneous stent placement in the left anterior descending (LAD) artery 14 years ago. The patient presented to the emergency department with a non-ST elevation myocardial infarction. Coronary angiography showed 90% mid-LAD stenosis, 90% mid-right coronary artery (RCA) stenosis and occlusion of the distal RCA, and 90% obtuse marginal (OM) branch stenosis. The left main artery was normal. Given the severity of his CAD, a coronary artery bypass grafting (CABG) surgery was planned.

Preoperatively, the patient was taking sitagliptin/metformin SR 100/2000 mg, amlodipine 5 mg, aspirin 100 mg, atorvastatin 40 mg, bisoprolol 2.5 mg, clopidogrel 75 mg, dapagliflozin 10 mg, lisinopril 5 mg, and pantoprazole 40 mg. All his medications were taken once per day. Clopidogrel was stopped 1 week before surgery. The preoperative carotid artery ultrasound doppler and routine blood investigations were all within normal limits. Preoperative transthoracic echocardiography is described in the Appendix.

The surgery was performed 2 weeks after the initial presentation to the emergency department. In the operating room, the patient was prepared and anesthetized in a routine manner. Intraoperative transesophageal echocardiography (TEE) was performed. (For a detailed account of the anesthetic, surgical, and TEE details, please see the Appendix). At the end of the procedure and on resuming mechanical ventilation and separation from the CPB, the patient’s airway pressure increased to 40 cm H_2_O. The lungs were over-distended and demonstrated little respiratory movement in response to mechanical ventilation. The initial recruitment maneuvers did not improve the situation. Suspecting bronchospasm, salbutamol puffs were administered, and ventilator parameters were adjusted to maintain the airway pressure below 40 cm H_2_O. Blood-tinged frothy secretions were observed in the endotracheal tube. Bronchoscopy confirmed the correct positioning of the endotracheal tube above the carina and revealed further frothy secretions in the airway, which were suctioned. The arterial blood gas (ABG) test at this stage showed a pH of 7.26, partial pressure of oxygen (PaO_2_) of 205 mmHg, partial pressure of carbon dioxide (PaCO_2_) of 55 mmHg, lactate of 1 mmol/L, SpO_2_ 99.8%, and HCO_3_ of 22.4 mmol/L. Furosemide (40 mg IV bolus, followed by 5 mg/hour infusion) was administered, along with additional salbutamol puffs through the endotracheal tube and a 200 mg IV bolus of hydrocortisone. Post-CPB, TEE showed mild dilatation of the right ventricle (RV) with bowing of the interatrial septum to the left. With diuresis and the institution of dopamine 5 mcg/kg/minute and norepinephrine 0.1 mcg/kg/minute, the RV pressure load improved, normalizing both interatrial septum and function. The ejection fraction (EF) was similar to the pre-CPB level, with no new regional wall motion abnormalities observed. All valve functions were normal, and the right ventricular systolic pressure was measured at 31 mmHg. The mode of ventilation was adjusted from volume-controlled to pressure-controlled, with an inspiratory pressure of 25 cm H_2_O and positive end-expiratory pressure (PEEP) of 5 cm H_2_O. Oxygenation was maintained, but the PaCO_2_ level in the ABG test was still high at 54 mm Hg; the ventilatory rate was increased accordingly. Protamine was administered following a test dose, and hemostasis was achieved. The initial attempt at sternal approximation resulted in compression of the distended lungs and a picture of acute tamponade. Sternal stenting and patch closure of the skin wound were then performed.

**Figure 1:**
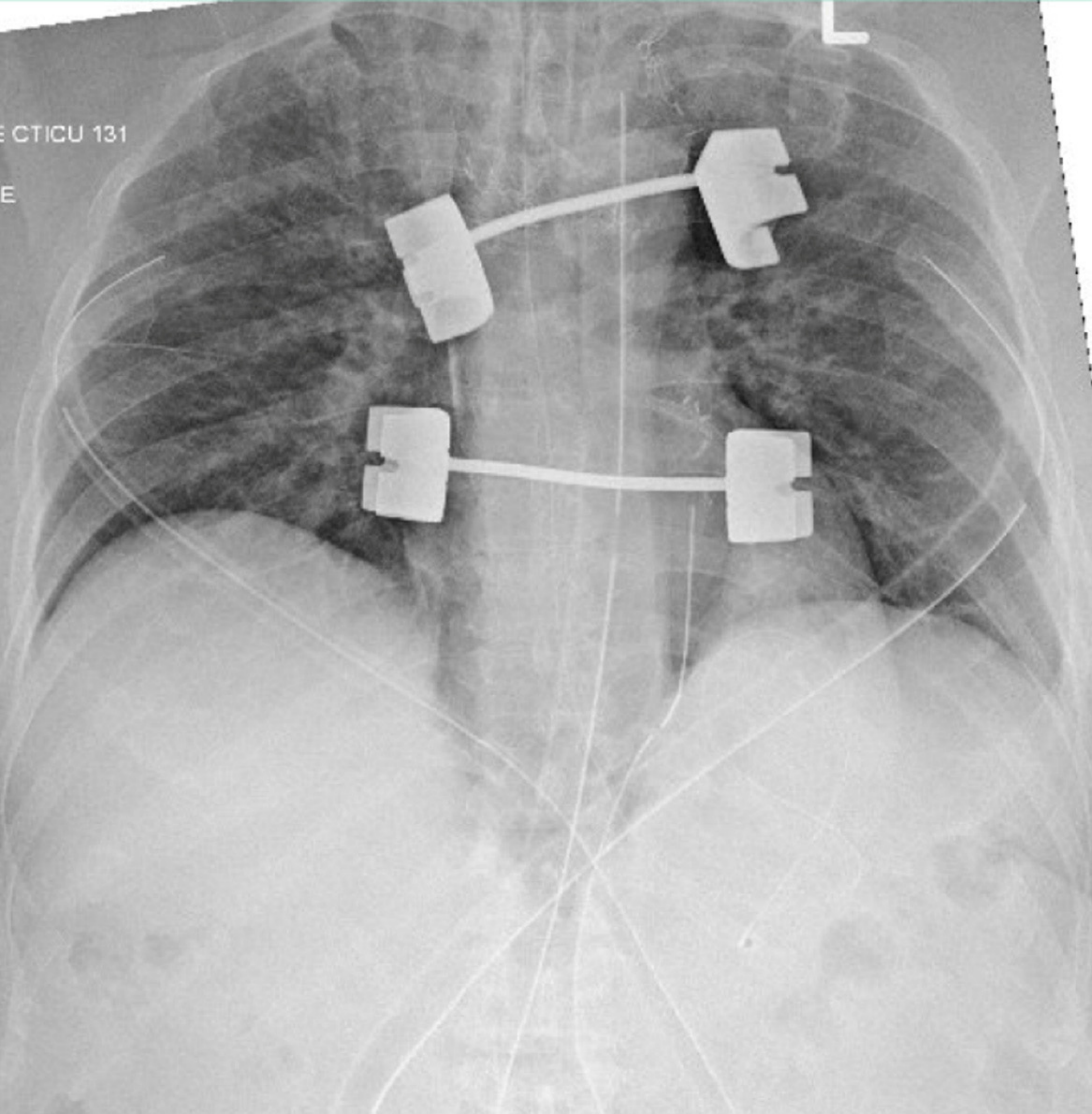
A chest roentgenogram taken in the ICU, showing pulmonary edema as well as sternal stents in situ.

In the intensive care unit (ICU), ventilator parameters were kept on pressure control with gradual improvement in oxygenation. A chest roentgenogram showed bilateral pulmonary congestion (Figure 1). The lung compliance gradually improved, and the patient remained intubated and ventilated, receiving sedation with fentanyl and propofol infusions alongside furosemide. The patient required norepinephrine, and dopamine infusions, which were continued until the chest closure where they were weaned, guided by hemodynamic monitoring. While on pressure-controlled ventilation with PEEP of 8 cm H_2_O and fractional inspired oxygen (FiO_2_) of 30%, the ABG test showed a PaO_2_ of 172 mmHg, PaCO_2_ of 39 mmHg, and oxygen saturation of 99.6%. The hematological and biochemical blood profiles remained within normal limits. However, troponin levels were initially 1110 ng/L, decreasing to 828 ng/L after 12 hours. Pro–B-type natriuretic peptide (Pro-BNP) levels were elevated at 3,289 pg/mL. Twenty-four hours later, the sternum was approximated without incident, the chest was closed in a routine fashion, and the patient was transferred to the general floor on the third postoperative day Upon discharge from the hospital, the patient did not have any residual complications of this event. The patient was followed in the outpatient clinic during the regular post-surgical visits, and he continued to do well without any marked consequences to the event.

## Case Discussion

This case presents a rare instance of REPE following routine CPB and CABG surgery. The diagnosis was supported by the exclusion of alternative differentials and the clinical presentation postoperatively. The differential diagnosis included cardiogenic pulmonary edema, upper airway obstruction, transfusion-related acute lung injury, transfusion-associated circulatory overload, protamine reaction, and bronchospasm.^4^ However, this patient had a normal EF at the end of the procedure and received neither protamine nor blood or its products prior to the development of pulmonary edema. The bronchoscopy confirmed a patent airway. Acute asthmatic attack and acute bronchospasm were ruled out by the shape of the capnography waveform and the presence of pulmonary edema.

REPE was reported in cases involving decompression of pneumothorax or lung re-expansion after prolonged lung collapse due to pleural effusion^5^ with an incidence of 1% and a mortality rate reaching 20%.^6^ The incidence of unilateral postoperative pulmonary edema in minimally invasive cardiac surgery with CPB is approximately 25%, but bilateral REPE, as seen in this case, is exceedingly rare, with an estimated incidence of less than 0.6%.^1,7–10^

The mechanism of REPE could be due to reperfusion injury secondary to free radicals’ generation.^11^ The release of proinflammatory cytokines and an increase in vascular permeability are also implicated in the pathogenesis of REPE.^11–14^ Altered surfactant function, potentially aggravated by CPB, is another potential mechanism.^12,15^ Prolonged CPB is associated with an increased risk of REPE.^3^ The case described above had a CPB time of 120 minutes. The relationship between prolonged CPB and postoperative complications is well-documented, although there is no agreement in the literature on when CPB is considered prolonged. In one study, a 30-minute increase in CPB time was shown to increase the odds of postoperative respiratory complications (odds ratio 1.17).^16^ The presence of chronic obstructive pulmonary disease, pulmonary hypertension, or right ventricular dysfunction are independent risk factors for the development of unilateral postoperative pulmonary edema.^14^

The use of diuretics, adjustment of ventilatory support, and careful hemodynamic monitoring were critical in managing the patient’s condition. It was hypothesized that protective lung ventilation during CPB can improve oxygenation and lung mechanics^17^ and can reduce the pulmonary inflammatory response to reduced perfusion.^18^ However, multiple randomized trials failed to show any statistically significant clinical benefits of lung ventilation during CPB compared to no ventilation in terms of postoperative complications, length of stay, or duration of ventilation.^19–21^ It should be noted that given the rare nature of REPE, clinical trials will not be able to detect any benefits of lung ventilation in the context of REPE, and therefore, these trials should be carefully interpreted in this context. The lungs were not ventilated during the CPB in this case. The successful management of such cases relies largely on a high index of suspicion, with the institution of immediate corrective measures. Further research into this unusual complication is warranted to improve prevention, early detection, and management strategies.

## Conclusion

This article describes a rare case of bilateral REPE following routine CPB in cardiac surgery. Despite a slightly prolonged ICU stay, the patient was discharged home without residual complications. Although rare, REPE should be considered in the differential diagnosis for acute pulmonary edema post-CPB, especially after excluding other common causes. The pathophysiological mechanisms thought to cause REPE include reperfusion injury with free-radical-mediated lung tissue damage, proinflammatory cytokine release, increased vascular permeability, and altered surfactant function. Management includes diuretics, ventilatory support, and hemodynamic monitoring. Further research is needed to determine the incidence, risk factors, and preventive strategies of the condition.

## Conflicts of interest

None.

## APPENDIX Preoperative transthoracic echocardiography:

– Ejection fraction (EF) of 43%, grade 1 diastolic dysfunction, and normal left atrial pressure.
– Hypokinesis in the mid-anterolateral, apical anterior, and apical lateral left ventricular wall segments, as well as in the mid-anterior, mid-anteroseptal, apical septal, and apical inferior were noted.
– No valvular lesions or pericardial abnormalities were noted.

## Intraoperative pre-CPB transesophageal echocardiography:

– Left ventricular EF was 50%.
– Grade 1 diastolic dysfunction, with no regional wall motion abnormalities.
– Good RV function, with an RV systolic pressure of 31 mmHg.
– The valvular function was normal.

## Conduct of anesthesia:

– A right radial arterial line was inserted under local anesthesia prior to induction.
– Induction was performed using midazolam 5 mg, fentanyl 250 μg, etomidate 10 mg, and rocuronium 100 mg.
– Endotracheal intubation was achieved under video laryngoscopy guidance.
– A right internal jugular central venous catheter was subsequently inserted.
– Anesthesia was maintained using inhalational agents, propofol, fentanyl, and cisatracurium.
– Cefazolin 2 g was administered after induction and again at the conclusion of the procedure.
– Tranexamic acid 2 g was given as a slow bolus at the beginning of surgery, followed by a 500 mg infusion over 4 hours.

## Surgical procedure

– Median sternotomy.
– Harvesting of saphenous vein and left internal thoracic artery.
– Total body heparinization.
– CPB at normothermia.
– The patient received two vein grafts to OM and RCA. The left internal thoracic artery was anastomosed to the LAD.
– Myocardial protection is achieved using intermittent cross-clamping and fibrillation.
– Cross-clamp time was 42 minutes.
– CPB time was 120 minutes.
